# Modeling employees' skills for sustainable banking services

**DOI:** 10.3389/fsoc.2022.985158

**Published:** 2022-12-05

**Authors:** Fariba Azizzadeh, Mohammad Shahidul Islam, Najeebah Naushin, Sebastian Zupok, Dariusz Soboń, Janusz Soboń, Ruslana Selezneva, Hamid Mohsin Jadah

**Affiliations:** ^1^Department of Management, Islamic Azad University, Piranshahr, Urmia, Iran; ^2^Brac Business School, Brac University, Dhaka, Bangladesh; ^3^Wyzsza Szkoła Biznesu National Louis University, Nowy Sącz, Poland; ^4^The Jacob of Paradies University, Gorzów Wielkopolski, Poland; ^5^Taras Shevchenko Kiev National University, Kyiv, Ukraine; ^6^College of Administration and Economics, University of Kerbala, Karbala, Iraq

**Keywords:** employees' skills, sustainable banking, honesty, patience, smartness, technological expertise, COVID-19

## Abstract

In Bangladesh, more clarity is needed on data that could contribute to the provision of sustainable banking services. Therefore, the qualitative exploration of bank employees' skills to advance contemporary banking careers and services has been rational. Moreover, limited knowledge of what constitutes a sustainable banking career and service inspires this study to adapt to the new normal post-COVID-19. Fifteen experienced employees from the banking industry participated in the interview to accomplish the research. The results from content data analysis showed that technical skills may vary from department to department based on employees' job responsibilities. However, the employee skills are more or less similar for different banks. The results further showed that the banking sector emphasizes the need for honesty from banks' employees, as they maintain a large amount of cash and other types of assets in their vaults. Additionally, the research participants expressed their sentiments regarding other skills, such as patience, smartness, and technological expertise. These skills are needed to carry out the day-to-day operations and achieve high customer satisfaction. Therefore, the study recommends that banks focus on creating an employee base with the skills found in the investigation to develop banking services.

## Introduction

The banking industry in Bangladesh requires highly skilled people who are considered the banks' assets. City Bank Limited is also highly dependent on its workforce to conduct its day-to-day activities smoothly and effectively. The banking industry in Bangladesh has changed enormously in recent years (Hossain, 2022). There was a time when people used to think that a bank existed to give loans to borrowers and keep deposits from its customers. However, nowadays, the concept of banking is different. The banks in Bangladesh have upgraded their activities and added more features to their services to create reliability and loyalty among their customers.

Additionally, the COVID-19 pandemic has changed necessary employee skills for the banking industry. Introducing the function of debit and credit cards, ATM banking, digital banking, and MIS activities in banking operations are some of the features the banks have added to their activities in recent years. As the activities of the banking industry have changed, the requirements for hiring bank employees have also changed (Charandabi, 2020; Khan et al., 2021; Yasmin et al., 2021). In traditional banking before COVID-19, most of the employee–customer interactions were performed face to face. After the recent pandemic, tasks that could be done remotely were done remotely (Azizzadeh and Hosseini, 2021). In addition, electronic banking received more attention (Alizadeh et al., 2020).

The technological expertise of organizations is provided by specialized human resources and cutting-edge technological equipment. Technological experts are necessary for organizations to work remotely. These specialists are experts in the latest equipment and technologies needed by organizations. Organizations need up-to-date technological experts in sufficient numbers to face the COVID-19 pandemic and its aftermath (Azizzadeh et al., 2013, 2016; Azizzadeh and Hosseini, 2021; Tavana et al., 2021).

Bank employees must communicate effectively in writing, negotiate successfully with clients, detect and predict problems, solve problems in groups, and discover speedy solutions to problems (Chowdhury, 2020). Moreover, Adebakin et al. (2015) evaluated skill requirements with a sample of 300 Nigerian employers. They discovered that information and communication technology, teamwork, decision-making, analytical (Abed Azad et al., 2022), and problem-solving skills were the most important. Furthermore, communication, adaptability, teamwork, and interpersonal skills were the most considerable talents employers needed in business graduates (Adebakin et al., 2015; Charandabi et al., 2021), according to McMurray et al. (2016). The latter evaluated graduate skill requirements with a sample of 72 businesses in Scotland (McMurray et al., 2016).

Honesty in organizations deserves unique attention as the workplace context adds further complexity (Derfler-Rozin and Park, 2022). People who score relatively high on honesty are sincere, fair, and humble (Greitemeyer, 2022). Public opinion polls consistently show that a person's occupation also affects how honest they are and that people are motivated to see themselves as honest for many reasons (Nault and Thau, 2022). Honesty and deception have unique precedents and consequences in social contexts. While modern professional standards emphasize honesty and the avoidance of deception, empirical work from workplaces shows that deception remains widespread (Hart, 2022).

Patience can bring more revenue to the service provider. The decision of whether to wait represents the inherent patience or the time to wait (Liu et al., 2022). Different cultures show different levels of patience. Countries belonging to the Germanic, Northern European, and Anglo/American cultural clusters show the highest levels of patience. Higher levels of individualism and lower levels of uncertainty avoidance also have a positive and significant correlation with the average patience of countries. Empirical research has also supported the prediction that greater wealth is associated with greater patience (Burro et al., 2022).

Smartness has recently emerged as a desirable feature of governments, cities, communities, infrastructures, people, and devices. There is no consensus on what “smartness” means and on how to identify its key components or dimensions. Some definitions highlight information technology and data, while others focus on sustainability (Nasr et al., 2021), openness, innovation, or flexibility (Gil-Garcia et al., 2016; Zupok, 2017). Smartness has far-reaching implications regarding expectations, identity development, and inequalities. Smartness is used as a mechanism of control and social status along racial and class lines (Hatt, 2012).

Although there have been several studies about the necessary employee skills for the banking sector, those studies need to include the impact of COVID-19 on the skill requirements of the banking industry. Apart from COVID-19, the changing activities of the banks have also created a broader scope to study more about the required skills for advancing contemporary banking careers and achieving sustainable banking services (Yasmin et al., 2021).

Research on sustainable banking services is still imprecise in Bangladesh. There is limited knowledge of practical skills for a sustainable banking career and service. Moreover, it is essential to know what changes are happening to the required employee skills after the emergence of COVID-19 and whether there are any changes. Therefore, it can be understood that the study holds significance for today's banks that are updated in their offerings and activities (Hossain et al., 2020; Yasmin et al., 2021). This study was conducted to explore the skills that the employees of the banking sector of Bangladesh must have. While the lack of data is a challenge, it is essential to learn about the changes in skill requirements of employees of banks as per the evolving banking operations to adapt to those changes and run banking activities smoothly.

Furthermore, the study will help the banks' employers choose the right people to carry out their operations effectively. Thus, it will help the banks reach their desired goals. Given that the nature of our current research seeks to explore the concepts of banking skills required in Bangladesh in depth, a qualitative approach was chosen.

The research points out the different functional activities of the bank. Moreover, the research analyzes how the bank is doing financially and whether it needs improvement or is doing more than enough. Furthermore, it provides a thorough industry and competitive analysis, which can help the bank identify its weaknesses and overcome them with effective and efficient strategies.

The research provides a brief idea about City Bank Limited and its different areas of functions. It shows the bank's different management tactics, such as leadership style, recruitment, selection procedure (SpriggHR, 2022), compensation system, and performance measurement. Moreover, it looks at the bank's financial performance, accounting practices, and marketing strategies (Ntayi et al., 2010; Siahaan et al., 2016; Black et al., 2022; Charandabi and Ghanadiof, 2022; Investopedia, 2022a,b).

Thus, based on the above background and research needs, the study has posed a broad research question: what skills do bank employees require to advance contemporary banking careers? More specifically, the study seeks to identify skills essential for advancing contemporary banking careers and to model bank employees' skills in achieving sustainable banking services.

The study can help City Bank Limited create a valuable employee base by finding the right employees' skills to provide excellent banking services to its customers and thus make the bank more sustainable and reliable in the financial industry in Bangladesh. Although City Bank Limited and other banks in Bangladesh have well-organized job descriptions and specifications, the changing banking activities have demanded a change in skill requirements. Moreover, the banks should have an employee base to tackle the challenges during the COVID-19 pandemic (Collegenp, 2022; Wikipedia, 2022a; Assignment Point, 2022b). For this reason, it is essential to identify whether the banks should update their job specifications to find the right people. Consequently, the study can be helpful for City Bank Limited and other Bangladesh banks to identify the right employees' skills for creating a valuable employee base for contemporary banking. Furthermore, the banks can attract more customers by providing outstanding service through their skilled employees, thereby achieving profitability and sustainability goals.

A total of 60 banks were founded in Bangladesh, of which 50 were private and 10 were public. Fifty-seven banks are in the commercial sector, and three are in the specialized category. Currently, nine foreign commercial banks operate in Bangladesh and 51 local banks (BanksBD, 2022).

Leaders in the banking industry are confronted with numerous huge obstacles when managing their talent. According to Quantum Workplace research, only 50% of employees in the banking sector are highly engaged, and 35% are a retention risk (Ryba, 2022). To retain key employees and maintain high employee engagement, we must identify employees who possess the necessary skills required in contemporary banking. On the other hand, the employees will get demotivated, which will impact their engagement in their work. On the other hand, a demotivated employee base will not be able to contribute much toward achieving the bank's goals and objectives. Thus, finding employee skills suitable for the banking sector is essential to gaining sustainable banking services (Kang and Lee, 2021; Zhong et al., 2021).

The skills of bank managers also lead to a better understanding of banking risks and increased access to capital-raising options. Improving financial expertise and skills in bank managers and employees is effective for managing bank capital. These skills of financial experts lead to a better understanding of banking risks. They lead to financing choices that facilitate the implementation of timely and less costly recapitalizations in the banks (Gilani et al., 2021). Improving communication skills is also essential to achieving maximum bank productivity (Chew, 2005).

A mismatch of skills with needs can be considered a cause of unemployment and an obstacle to economic development (Chomać-Pierzecka et al., 2022; SpriggHR, 2022). In addition, the consequences in society at the micro level and the enterprise level are also considered harmful (Adely et al., 2021). As a result, it is an issue that banks should consider.

Human resources are the main resource in organizations, so the remaining assets would only be worth a little with them. The level of expectations of employees at Dey Bank and the realities they are confronted with is a topic that one researcher addressed. To reduce the gap between expectations and facts, in addition to improving the level of salaries and benefits, improving the bank employee's mental health and physical condition is one of the recommended factors. These factors should be considered to improve bank employees' skills (Azizzadeh et al., 2022; Assignment Point, 2022a).

Employees are assets for an organization, and without such human assets, an organization cannot operate effectively and efficiently. Moreover, employees with no relevant skills and abilities cannot be considered assets; instead, they become a burden to the organization as the firm has to pay them, which is a cost or expense the firm has to incur. In other words, it can be said that the company is investing in assets that will not provide them with any return. Thus, ensuring the firm has the right skills and abilities is mandatory. In the same way, City Bank Limited and other banks should know what skills and abilities are suitable for them, the employee skills they need more of, and so on. With proper research on necessary employee skills, it is possible to find the right human assets (Torre Olmo et al., 2021).

Moreover, if employees with the desired skills cannot be recruited, it will eventually hamper employee engagement and motivation. For example, if banks hire employees with irrelevant skills, they will find their jobs monotonous, and soon, they will lose their patience and feel frustrated with their work. Furthermore, such demotivation and indifference toward work will hamper their productivity, as they will not focus on their tasks. As a result, the banks will suffer losses due to inconvenience in their daily operations (Yusuf and Ichsan, 2021).

Employees should explore their careers in sectors where they find a good fit for their skills and that sector. The concept of person-job fit can be included here as well. In other words, the compatibility between persons and the job or tasks they undertake is known as person-job fit. The employees should choose an area of their expertise. Otherwise, they will not enjoy their work. Instead, they will feel less engaged in their work, negatively affecting their productivity (Kang and Lee, 2021).

Moreover, getting into an organization that matches the employees' skills is also vital. Employees who cannot focus on their jobs will not perform the assigned tasks, hindering their careers. Without performing well, employees cannot accomplish growth in their careers. The same logic applies to the banking industry. People who want to work in the banking industry should possess relevant skills in the banking sector. Otherwise, they will not witness career growth or success (Zhong et al., 2021).

The expansion of COVID-19 has caused great concern internationally among organizations. The virus has jeopardized the fundamental interests of organizations and forced them to take a series of special actions. Many organizations have been influenced by the COVID-19 pandemic (Azizzadeh and Hosseini, 2021). The COVID-19 pandemic has introduced working from home and virtual spaces (Azizzadeh et al., 2013, 2016) to the corporate world. Though people were aware of freelancing, the COVID-19 pandemic has forced organizations to allow employees to work from home to ensure employee safety. The banking industry in Bangladesh also faced massive pressure due to the pandemic, as people started coming to banks more frequently to withdraw money. During the pandemic, many employees lost their jobs, and in some cases, they were getting lower salaries than the industry standard. As a result, they were running out of savings (Naeem and Ozuem, 2021).

**Figure 1:**
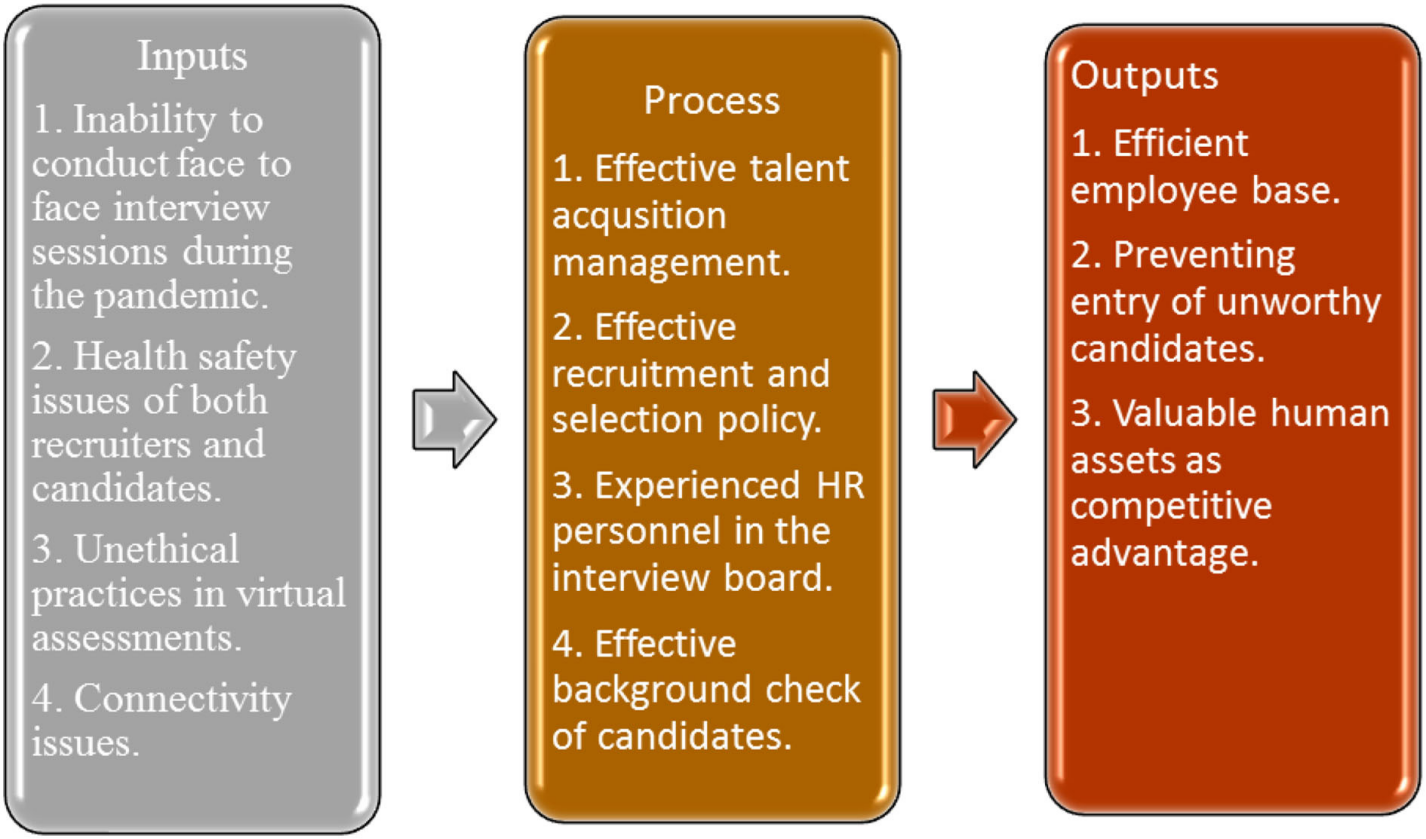
Recruitment problems and solutions in banks during COVID-19.

Moreover, it took much work for the banks to continue recruiting the right employees due to the restrictions of COVID-19. As a result, some banks failed to choose candidates with relevant skills, affecting their day-to-day activities and thus affecting their organizational objectives (Yusuf and Ichsan, 2021). The summarized literature on the HRM approach in the bank during COVID-19 is demonstrated in Figure 1.

## Methodology

The study explored the skills essential for advancing contemporary banking careers and designed a model of bank employees' skills in achieving sustainable banking services. The study adopted the qualitative data collection approach to gather and evaluate non-numerical information to comprehend concepts, ideas, or experiences (Hennink et al., 2020). Moreover, the research contained descriptive information from people working in the banking sector and drew conclusions from information. The study followed a qualitative approach to gathering information (Panfilova et al., 2019).

One of the researchers conducted the observation while working at City Bank Limited as an intern. Moreover, some parts of the report were created with the help of secondary data available from various sources. This research was conducted mainly using primary data. First, a questionnaire (Appendix) including ten questions was prepared for conducting interview sessions. Moreover, some interviews were conducted through phone calls, and some were conducted in person. This way, the raw data were gathered (WallStreetMojo, 2022), and the most relevant and valuable information was extracted through content data analysis (Blogspot, 2022).

The study is conducted by adopting non-probability sampling, incorporating non-random selection based on convenience or other criteria, which allows us to collect data quickly. More specifically, the results are prepared using convenience sampling, as the sample includes people who are the easiest to reach. The target people for asking questions are not random people. They are employees already working in banks in various positions. As banking people always remain busy due to immense work pressure, only easy-access individuals are taken for the interview (Abedi Jafari et al., 2011).

The sample is selected based on some criteria. First, participants who have worked or are already working in the banks of Bangladesh were recruited for interviews. Moreover, participants from officer to management positions are taken for the study as their information is more relevant and valuable. Fifteen employees from the banking industry in Bangladesh are taken to conduct interview sessions through phone calls and in person to prepare the study's findings.

The 15 employees from the banking industry, from officers to management positions, were contacted by phone first to determine their convenient time. Before starting the interview session, a brief idea about the study and its importance was presented to them. Furthermore, they were assured of their confidentiality and anonymity. The study required recording the interview sessions to deliver results properly. Nevertheless, some participants refused to allow the session to be recorded because they were worried about information getting leaked or their identity being exposed.

This study aimed mainly to model employees' skills for sustainable banking services. Hence, the data were needed to reduce the sentiments of interviews into defined categories for enhanced interpretation (Zhang et al., 2019). Reasonably, this study's data analysis process is directed as the content data analysis. The transcribed interviews are read and analyzed three times, considering “what is said?” and “why is said?” These questions help us engage in data and extract interrelated senses (Yusuf and Ichsan, 2021).

## Results and discussion

The purpose of analyzing the content of the text, interview, document, etc., is to identify the author's or interviewee's goals, values, culture, and desires. In other words, in content analysis, cognition is the subconscious of the text and its owner (Freud, 1989). The purpose of content analysis is not only to describe the features of the text but, more importantly, to conclude and deduce the content of the thinkers (Abedi Jafari et al., 2011; Azizzadeh, 2019). Qualitative content analysis can be considered a research method for the subjective interpretation of textual data's content through systematic classification, coding, and theme building or the design of known patterns (Hsiu-Fang and Sieh Shannon, 2005). Qualitative content analysis allows researchers to interpret the authenticity and truth of data in a subjective but scientific process. The objectivity of the results is guaranteed by a systematic coding process. Qualitative content analysis goes beyond texts' words or objective content and tests themes or patterns that are overt or covert as overt content (Zhang, 2006).

The steps of content analysis in a qualitative research framework are as follows: 1. text analysis and description, 2. describing and interpreting the text, and 3. text composition and integration. Familiarity with the text, generating initial codes and coding, and then searching for and understanding themes are the main tasks in the text analysis step. We drew a grid of themes and analyzed themes in the second step. Finally, we reported the findings in the third step (Azizzadeh, 2019).

The coding process includes three stages (open, axial, and selective). In the coding stages, appropriate codes were assigned to different parts of the data at the open coding stage. Then, researchers in the axial coding stage pioneer pivotal encryption by thinking about the different dimensions of these parts and finding links between them. Finally, they explored the categories through selective encryption. They eventually created a theoretical framework that included main themes and sub-themes (Azizzadeh, 2019; Azizzadeh and Pourranjbar, 2021).

In the data analysis section, researchers attempted to reduce the possibility of their implied and unspoken meanings by immersing themselves in the obvious meanings of the words and sentences produced. Therefore, after reviewing the obtained data, open coding began, and the data were categorized based on the logic of this coding. At this stage, the data were separated from each other, and concepts and sub-themes emerged from them. In the next stage, axial coding was started. The concepts and sub-themes separated in the previous stage were connected in a new combination based on their relationships with other sub-themes and concepts. In the final stage, the coded data were grouped into themes.

By analyzing the data, the main themes in this research were identified (Table 1). A total of three main themes and 13 sub-themes were extracted from the data analysis after coding steps on them. They included the importance of staff skills, the impact of these skills, and organizational outcomes. Employees' skills should include patience, smartness, excellent communication skills, and adequate technological expertise. These themes have an effect on attracting and retaining talented workers, boosting productivity and efficiency inside organizations, and lowering the rate of employee turnover. Organizational outcomes if this process included three subthemes. They were “achieving sustainable banking services,” “increased profitability and growth,” and “the advancement of a banking career.”

**Table 1:** Coding and theme forming.

**Row**	**Main theme**	**Sub-themes**
1	Necessary employee skills	1. Honesty
		2. Excellent communication skills
		3. Adequate technological expertise
		4. Smartness
		5. Patience
2	Impact of these skills	6. Right employees found
		7. Increased employee productivity
		8. Less employee turnover
		9. Increased employee retention
		10. Creation of a valuable employee base
3	Organizational outcome	11. Achieving sustainable banking services
		12. Increased profitability and growth
		13. Advancement of banking career

The study's main goal was to highlight the skills essential for advancing contemporary banking careers and to model bank employees' skills in achieving sustainable banking services. To conduct the study, we gathered qualitative information from 15 participants working at different banks in Bangladesh. Among them, some informants provided a wide range of data, as they were nonchalant during the conversation. On the other hand, some informants seemed slightly nervous and appeared anxious about maintaining their job security. Most informants talked about honesty as a skill for the people working at banks, and they showed logic. For example, one of the informants said, “A banker should be honest, and it is not important whether he is a support staff member or a manager. You see, banks keep a high amount of reserves, and we need to answer to the Bangladesh Bank for everything, and if they find any discrepancy, the banks can face many hassles. Therefore, honesty should be there at all levels of a bank.” Furthermore, they talked about having excellent communication skills. For example, an employee said, “a person who works at a bank should have excellent communication skills because customers can be from rural areas and cities. They must be handled effectively; it is impossible without a smooth communication flow.”

**Figure 2:**
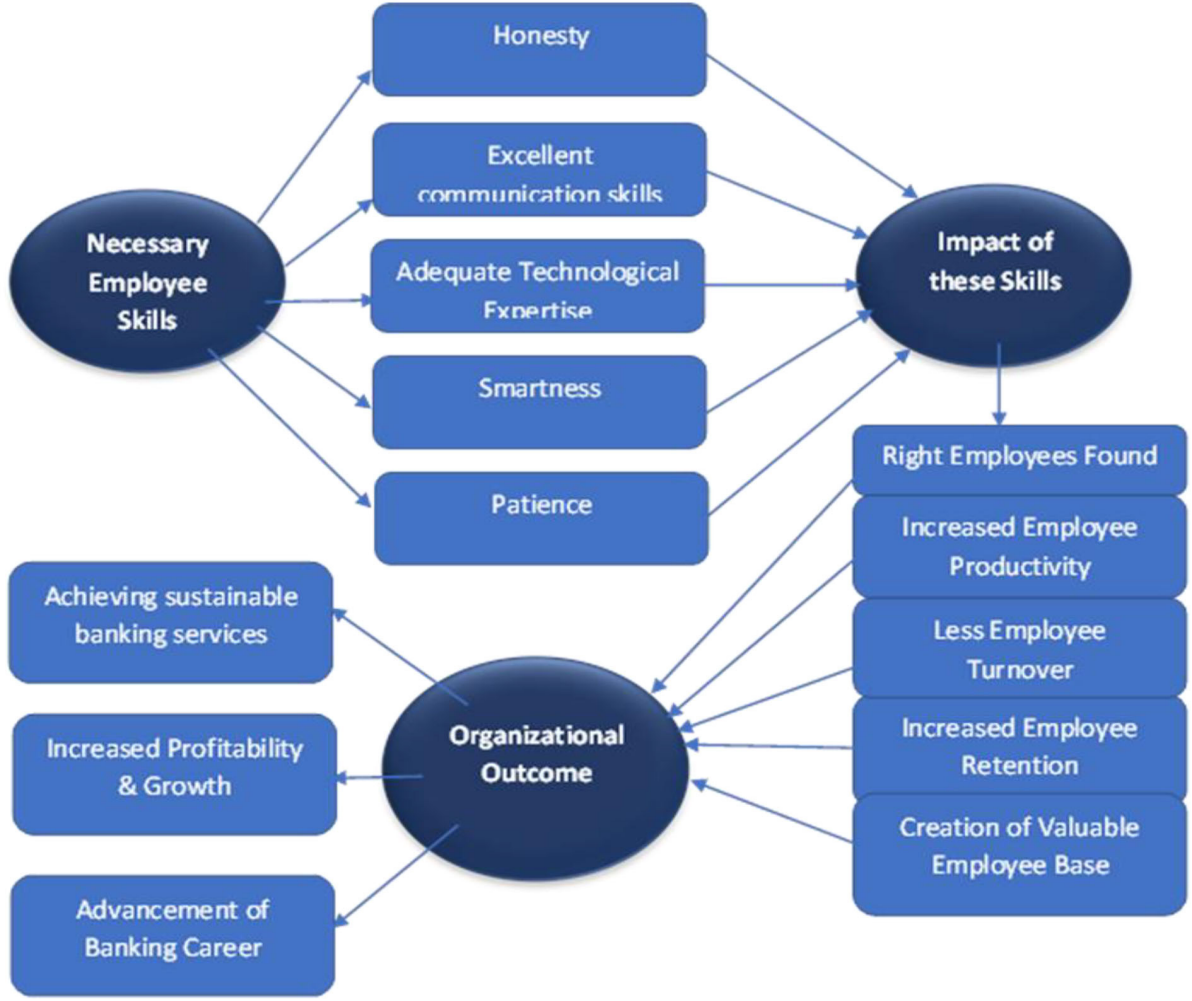
Models of bank employees' skills in achieving sustainable banking services.

Furthermore, they identified soft skills like patience and smartness. After COVID-19, it has become essential to have the technological expertise to work from home effectively. Some informants said that the skill requirements stayed mostly the same after COVID-19. For example, one said, “It's not like customers are not coming to banks; rather, they are coming more frequently after the pandemic as they are running out of their savings, and it is creating a huge pressure on employees working at the cash department and the customer service department. Therefore, I do not see any major change in skill requirements after COVID-19.” Therefore, it can be seen that the informants varied in providing data. After analyzing the information provided by the informants, it can be identified that banking practitioners should have honesty, excellent communication skills, adequate technological expertise, smartness, and patience at all levels (Figure 2).

As seen in the model (Figure 2), most informants have said that the first quality the banks' employees are expected to show is their honesty. Though banking operations have changed over time, it has not changed that a bank is a giant reserve of money, and the people who work in banks should be honest to keep the reserve of money safe and secure. Secondly, communication skills are essential. A banking employee should communicate with all types of people and bring business to the bank. Without communication, there is always a gap in the process, whether it is the human resources division, retail banking, or any other bank division.

Furthermore, a banking employee should be tech-savvy in this era of technological advancement and during the COVID-19 pandemic. They should have a firm grip on computer skills like Microsoft PowerPoint, and Microsoft Excel. Banking activities have expanded over time. It is not like sitting and calculating with some paper and a pen. Knowledge of Microsoft Excel is crucial for a person who works at a bank today to create reports and keep records. Employees should have the technical expertise to work from home. Moreover, banks prefer innovative individuals nowadays. It is not enough if a person is good at academics; they should also be intelligent, sensible, and easily adaptable. Educational background is essential, but private commercial banks like City Bank Limited prefer a complete package of qualities within an individual. Patience is another quality the banks want in their employees. Despite getting updated over time, most banking procedures are lengthy and time-consuming and should be handled patiently.

The banks also train their employees to fill the skills gap and keep them motivated. Some banks have their own training centers to provide lecture-based training, and some offer on-the-job training programs. Sometimes, the senior employees play the role of mentors for the junior employees. To maintain employee motivation, banks take several initiatives. For example, City Bank Limited provides “Thank You” cards to employees who perform better, thus keeping them motivated. Moreover, the bank arranges a “Rewards and Recognition Ceremony” to reward its employees by signifying their contribution to the success of City Bank Limited.

## Conclusion

The banking sector in Bangladesh requires highly skilled people who are considered the banks' assets. City Bank Limited is also highly dependent on its workforce to conduct its day-to-day activities smoothly and effectively (Wikipedia, 2022a). There needs to be more knowledge of practical skills for a sustainable banking career and service. Research on sustainable banking services still needs to be clarified in Bangladesh. Moreover, it is essential to know what changes are happening for the required employee skills after the emergence of COVID-19 (Azizzadeh and Hosseini, 2021) and whether there are any changes. Consequently, the study can be helpful for City Bank Limited and other Bangladesh banks to identify the right employees' skills for creating a valuable employee base for contemporary banking. Furthermore, the banks can attract more customers by providing outstanding service through their skilled employees, thereby achieving profitability and sustainability.

Thus, City Bank Limited should hire people who fulfill the skills revealed in the results. Furthermore, the bank can modify its job description and specifications by conducting a more in-depth job analysis. The bank's employees can be its human assets and contribute to achieving its goals. The banking industry in Bangladesh has been struggling for a long time, and the COVID-19 pandemic has worsened the situation. Thus, banks like City Bank Limited need to focus on increasing efficiency and profitability, and a valuable employee base with the essential skills can contribute to achieving such goals. Therefore, this study helps determine the suitable abilities for bankers to gain sustainability in banking services.

The theoretical implication of this study is noteworthy. The study theorizes that selecting suitable employees for an organization through identifying and fostering employee skills essential to that organization's genre leads to various positive outcomes. For example, our explored model (Figure 2) indicates that the necessary and relevant employee skills increase employee engagement, productivity, and retention. In addition, it identifies that people who choose work areas that are pertinent to their abilities have a greater chance of achieving success in their careers. Thus, this study advances the extant literature on HRM in the banking sector (Management Study Guide, 2022; SpriggHR, 2022; Wikipedia, 2022b) by understanding bank employees' skills in achieving sustainable banking services and professions.

The authors should discuss the results and how they can be interpreted with the previous studies with the working hypotheses in mind. The findings and their implications should be addressed in the broadest possible context. Future research directions may also be highlighted.

The study also has important practical implications for bankers/practitioners (e.g., managers, aspirants, entrepreneurs) in addition to its theoretical implications.

First, the banks of Bangladesh, like City Bank Limited, can learn about the skills essential to the banking industry and apply the findings of the study to their recruitment (SpriggHR, 2022; Wikipedia, 2022b). For example, the banks can include assessments to identify whether candidates have those skills in their recruitment phases. In this way, the study's findings can be utilized in the real-life recruitment procedures of banks.

Second, the study results can benefit people who want to establish their careers in the banking industry (Assignment Point, 2022b). As the study found out the skills essential to the banking industry, the candidates who want to explore their careers can know whether they possess such skills and are suitable for working at banks. It will help them choose the right path for building their career.

Third, the results will help bank practitioners modify their job descriptions to attract the right pool of candidates. Thus, it will allow banks to find suitable candidates. Suitable employees eventually become human assets for banks, which will help the banks achieve a competitive advantage over competitors in the market.

Finally, if banks can create a solid employee base with the help of the study results, the entire banking industry in Bangladesh will benefit. Bangladesh's financial sector struggled before the pandemic, and the pandemic worsened the situation. In these circumstances, if banks can attract and hire people possessing the skills mentioned in the study, the banking industry in Bangladesh can experience a positive change in its profitability and growth, which will help stabilize the unsteady economy of the country.

We must consider that this research has identified the appropriate and required banking skills in Bangladesh. As a result, it is recommended that banks strengthen these skills in their employees. In this way, banks can attract more customers and increase their profitability. The use of outside psychologists or the recruitment of organizational psychologists can be considered to bolster these abilities. It is also recommended that we select people who have these skills when hiring new staff. It is recommended that these items be considered in the banks' policies.

The study suggests that a mixed method may provide a clear picture of the study objectives. Thus, a future research attempt based on a mixed method is welcome. In contrast, future research can include studying employee skills specific to separate departments within a bank. This can help the banks hire more suitable employees because backend and frontend employees may be different. Moreover, the skill requirements before and after the COVID-19 pandemic should be studied theoretically in the quantitative dimension (Arabi Belaghi et al., 2016; Valizadeh Gamchi et al., 2019) with a larger sample size. The selection of attributes for different perspectives by using different intelligent statistical tools and studying the level and requirement of different aptitudes or behavioral indicators can be made to achieve a more quantified and visible outcome, which is something researchers are recommended to address in the future. Additionally, to show how many users used electronic services before COVID-19 and how many use them now, we suggest that other researchers fill this gap in the literature.

The research could have more details and information if there were no time constraints and confidentiality issues. Although the study provides valuable insights into the employee skills essential for advancing contemporary banking careers and models bank employees' skills in achieving sustainable banking services, the study is not without limitations. First, the sample size needed to complete the study interview was small (e.g., fifteen participants). Second, the study could have included more information if the sample size had been significantly larger to evaluate the data saturation. Moreover, the time to complete the research was insufficient, and the latent data still exist and need to be explored further. Third, the respondents for the study were full-time workers at banks, and they could not give enough time, and some questions were answered in a hurry. Finally, the study does not identify skills for specific areas within banks; it only talks about common skills in the banking industry. Conducting other research with a qualitative approach and using other methods, such as grounded theory and thematic analysis or network analysis by researchers in this field, can be useful.

## Ethics statement

Ethical review and approval was not required for the study on human participants in accordance with the local legislation and institutional requirements. Written informed consent for participation was not required for this study in accordance with the national legislation and the institutional requirements.

## Author contributions

FA and MI: conceptualization. FA: methodology, writing—review and editing, and project administration. MI, NN, and SZ: validation. NN and FA: formal analysis. NN: investigation and visualization. DS, JS, RS, and HJ: resources. MI and NN: writing—original draft preparation. MI: supervision. All authors have read and agreed to the published version of the manuscript.

## Conflict of interest

The authors declare that the research was conducted in the absence of any commercial or financial relationships that could be construed as a potential conflict of interest.

## Publisher's note

All claims expressed in this article are solely those of the authors and do not necessarily represent those of their affiliated organizations, or those of the publisher, the editors and the reviewers. Any product that may be evaluated in this article, or claim that may be made by its manufacturer, is not guaranteed or endorsed by the publisher.

## Appendix

**Table:**

**Interview questions**
Here are the questions for the interview sessions provided below: Do you have any idea about contemporary banking? Do you think there is a relationship between employee skills and banking services? What are the qualities/skills that a bank looks for in its employees? How did skills requirements change post-COVID-19? How did the banks' operations transform in recent years? How can employees contribute to making a bank sustainable? How do a bank's resources influence an employee's skills and productivity? What does a bank do to fill up the employee skills gap? Do you think the banks' employees require training or skill development programs to make banks better sustainable? How do long working hours in the banking industry affect employee motivation and employee skills?

## References

[B1] Abed Azad F. Ansari Rad S. Hairi Yazdi M. R. Tale Masouleh M. Kalhor A. (2022). Dynamics analysis, offline–online tuning and identification of base inertia parameters for the 3-DOF Delta parallel robot under insufficient excitations. Meccanica 57, 473–506. 10.1007/s11012-021-01464-7

[B2] Abedi Jafari H. Taslimi M. Faghihi A. Sheikhzadeh M. (2011). Content analysis and content analysis: a simple and efficient way to explain patterns in qualitative data. J. Strategic Manage. Thought 2, 151–198.35777803

[B3] Adebakin A. B. Ajadi T. O. Subair S. T. (2015). Required and possessed university graduate employability skills: perceptions of the Nigerian employers. World J. Educ. 5, 115–121. 10.5430/wje.v5n2p115

[B4] Adely F. I. J. Mitra A. Mohamed M. Shaham A. (2021). Poor education, unemployment and the promise of skills: the hegemony of the “skills mismatch” discourse. Int. J. Educ. Dev. 82, 102381. 10.1016/j.ijedudev.2021.102381

[B5] Alizadeh A. Chehrehpak M. Nasr A. K. Zamanifard S. (2020). An empirical study on effective factors on adoption of cloud computing in electronic banking: a case study of Iran banking sector. Int. J. Bus. Inform. Syst. 33, 408–428. 10.1504/IJBIS.2020.105833

[B6] Arabi Belaghi R. Valizadeh Gamchi F. Bevrani H. (2016). Likelihood based inference on progressive type-II hybrid-censored data for burr type III distribution. Reliability Theory Appl. 194, 194–199.

[B7] Assignment Point (2022a). Human Resource Management Practices of the City Bank Limited. Available online at: https://www.assignmentpoint.com/business/human-resource-management/human-resource-management-practices-of-the-city-bank-limited.html (accessed January 22, 2022).

[B8] Assignment Point (2022b). Report on Banking Activities Analysis of the City Bank Limited. Available online at: https://www.assignmentpoint.com/business/finance/report-on-banking-activities-analysis-of-the-city-bank-limited.html (accessed January 22, 2022).

[B9] Azizzadeh F. (2019). Pathology of tourist attraction problems in St. Mary Church of Urmia. J. Environ. Manage. Tour. 8, 1956–1962. 10.14505//jemt.v10.8(40).25

[B10] Azizzadeh F. Babapour H. Akhavan M. Azizzadeh S. Khatoon V. D. T. Hosseini A. (2022). Expectations and organizational realities: the relationship between person and organization. Int. J. Interdiscip. Org. Stud. 17, 23–34. 10.18848/2324-7649/CGP/v17i01/23-34

[B11] Azizzadeh F. Hosseini A. (2021). The impact of COVID-19 on public organizations. J. Contemp. Res. Bus. Adm. Econ. Sci. 1, 64–67. 10.52856/jcr311280119

[B12] Azizzadeh F. Latifi Y. Azizzadeh S. (2016). Virtual communities of police service support: providing SWOT model in line with the order and security. Online J. Commun. Media Technol. 6, 166–180. 10.29333/ojcmt/2575

[B13] Azizzadeh F. Pourranjbar S. (2021). The causes of discharge against medical advice and suggestions for its reduction in Tabriz Sina Medical Center (phenomenological study). Indian J. Med. Sci. 73, 88–92. 10.25259/IJMS_64_2020

[B14] Azizzadeh F. Shirvani A. R. Sarihi Sfestani R. (2013). Public service support through the virtual social networks. Manage. Adm. Sci. Rev. 2, 293–303.

[B15] BanksBD (2022). All Bank Listings in BD. Available online at: https://www.banksbd.org/banks (accessed November 22).

[B16] Black S. Gardner D. Pierce J. Steers R. (2022). Performance Appraisal Systems. Available online at: https://opentextbc.ca/organizationalbehavioropenstax/chapter/performance-appraisal-systems/ (accessed January 25, 2022).

[B17] Blogspot (2022). Management Functions and Organizational Behavior of the City Bank Limited, Bangladesh. Available online at: http://majorstudy.blogspot.com/2015/09/management-functions-and-organizational.html (accessed January 22, 2022).

[B18] Burro G. McDonald R. Read D. Taj U. (2022). Patience decreases with age for the poor but not for the rich: an international comparison. J. Econ. Behav. Org. 193, 596–621. 10.1016/j.jebo.2021.11.005

[B19] Charandabi S. Ghanadiof O. (2022). Evaluation of online markets considering trust and resilience: a framework for predicting customer behavior in E-commerce. J. Bus. Manage. Stud. 4, 23–33. 10.32996/jbms.2022.4.1.4

[B20] Charandabi S. E. (2020). Prediction of customer churn in banking industry. Age 18, 38–92. 10.13140/RG.2.2.24177.56167

[B21] Charandabi S. E. Ghashami F. Kamyar K. (2021). US-China tariff war: a gravity approach. Bus. Econ. Res. 11, 69–77. 10.5296/ber.v11i3.18757

[B22] Chew K. S. (2005). An investigation of the English language skills used by new entrants in banks in Hong Kong. English Specific Purposes 24, 423–435. 10.1016/j.esp.2005.02.004

[B23] Chomać-Pierzecka E. Sobczak A. Urbańczyk E. (2022). RES market development and public awareness of the economic and environmental dimension of the energy transformation in Poland and Lithuania. Energies 15, 5461. 10.3390/en15155461

[B24] Chowdhury F. (2020). Skills Gap of Business Graduates in the Banking Sector of Bangladesh: Employers' Expectation Versus Reality. Available online at: https://www.researchgate.net/publication/346426946_Skills_Gap_of_Business_Graduates_in_the_Banking_Sector_of_Bangladesh_Employers%27_Expectation_Versus_Reality (accessed January 22, 2022).

[B25] Collegenp (2022). Top 13 Essential Basics of Banking Skills | Collegenp. Available online at: https://www.collegenp.com/article/basics-of-banking-skills/ (accessed January 22, 2022).

[B26] Derfler-Rozin R. Park H. (2022). Ethics and honesty in organizations: unique organizational challenges. Curr. Opin. Psychol. 47, 101401. 10.1016/j.copsyc.2022.101401 35878580

[B27] Freud S. (1989). Introduction a La Psychanalyse. Paris: Petit Bibliotheque Payot.

[B28] Gilani U. Keasey K. Vallascas F. (2021). Board financial expertise and the capital decisions of US banks. J. Corporate Finance 71, 102091. 10.1016/j.jcorpfin.2021.102091

[B29] Gil-Garcia J. R. Zhang J. Puron-Cid G. (2016). Conceptualizing smartness in government: an integrative and multi-dimensional view. Government Inform. Q. 33, 524–534. 10.1016/j.giq.2016.03.002

[B30] Greitemeyer T. (2022). Honesty-humility, the dark tetrad, and ideological beliefs: their incremental validity in predicting explicit prejudice toward asylum seekers. Personality Individual Diff. 197, 111786. 10.1016/j.paid.2022.111786

[B31] Hart J. L. (2022). Deception, honesty, and professionalism: a persistent challenge in modern medicine. Curr. Opin. Psychol. 47, 101434. 10.1016/j.copsyc.2022.101434 35998527

[B32] Hatt B. (2012). Smartness as a cultural practice in schools. Am. Educ. Res. J. 49, 438–460. 10.3102/0002831211415661

[B33] Hennink M. M. Hutter I. Bailey A. (2020). Qualitative Research Methods, 2nd Edn. Sage.

[B34] Hossain A. Humayun K. Chowdhury M. Hasan S. Shamsuzzaman M. Fahim A. Y.. (2020). Banking service in Bangladesh: the impact of service marketing mix on purchase intention of university students. Strategic Change 29, 363–374. 10.1002/jsc.2335

[B35] Hossain F. (2022). Employee Job Satisfaction of the City Bank Limited. Available online at: https://www.academia.edu/19099517/Employee_Job_Satisfaction_of_The_City_Bank_Limited (accessed January 22, 2022).

[B36] Hsiu-Fang H. Sieh Shannon S. E. (2005). Three approaches to qualitative content analysis. Qual. Health Res. 15, 1277–1288. 10.1177/1049732305276687 16204405

[B37] Investopedia (2022a). Debt-to-Equity (D/E) Ratio. Available online at: https://www.investopedia.com/terms/d/debtequityratio.asp (accessed January 25, 2022).

[B38] Investopedia (2022b). Everything Marketing Entails. Available online at: https://www.investopedia.com/terms/m/marketing.asp#:~:text=What%20Is%20Marketing%3F,on%20behalf%20of%20a%20company (accessed January 25, 2022).

[B39] Kang E. Lee H. (2021). Employee compensation strategy as sustainable competitive advantage for HR education practitioners. Sustainability 13, 1049. 10.3390/su13031049

[B40] Khan A. G. Lima R. P. Mahmud M. S. (2021). Understanding the service quality and customer satisfaction of mobile banking in Bangladesh: using a structural equation model. Global Bus. Rev. 22, 85–100. 10.1177/0972150918795551

[B41] Liu J. Chen J. Bo R. Meng F. Xu Y. Li P. (2022). Increases or discounts: price strategies based on customers' patience times. Eur. J. Operat. Res. 305, 722–737. 10.1016/j.ejor.2022.06.015

[B42] Management Study Guide (2022). What Is Human Resource Planning? Available online at: https://www.managementstudyguide.com/human-resource-planning.htm (accessed January 25, 2022).

[B43] McMurray S. Dutton M. McQuaid R. Richard A. (2016). Employer demands from business graduates. Educ. Train. 58, 112–132. 10.1108/ET-02-2014-001736056552

[B44] Naeem M. Ozuem W. (2021). The role of social media in internet banking transition during COVID-19 pandemic: using multiple methods and sources in qualitative research. J. Retail. Consum. Serv. 60, 102483. 10.1016/j.jretconser.2021.102483

[B45] Nasr A. K. Tavana M. Alavi B. Mina H. (2021). A novel fuzzy multi-objective circular supplier selection and order allocation model for sustainable closed-loop supply chains. J. Clean. Prod. 287, 124994. 10.1016/j.jclepro.2020.124994

[B46] Nault K. A. Thau S. (2022). Professions, honesty, and income. Curr. Opin. Psychol. 47, 101403. 10.1016/j.copsyc.2022.101403 35872470

[B47] Ntayi J. M. Munene J. C. Eyaa S. (2010). Salesforce behavioural performance of accounts relationship managers (ARMS) in Uganda's commercial banks: a qualitative analysis. J. Retail Leisure Property 9, 5–23. 10.1057/rlp.2009.19

[B48] Panfilova E. E. Borisova V. V. Demidov L. N. Ushanov A. E. Maramygin M. S. (2019). The assessment and management of credit risk of commercial banks. Opcion 35, 613–627.

[B49] Ryba K. (2022). How to Improve Employee Engagement in the Banking Sector. Available online at: https://www.quantumworkplace.com/future-of-work/employee-engagement-in-banking-sector (accessed January 22, 2022).

[B50] Siahaan E. Gultom P. Lumbanraja P. (2016). Improvement of employee banking performance based on competency improvement and placement working through career development (case study in Indonesia). Int. Bus. 10, 255–261. Available online at: https://dupakdosen.usu.ac.id/bitstream/handle/123456789/58139/Improvement%20of%20Employee%20Banking%20Performance%20Based....pdf?sequence=6

[B51] SpriggHR (2022). What Is the Difference Between Recruitment and Selection? Available online at: https://sprigghr.com/blog/hr-professionals/what-is-the-difference-between-recruitment-and-selection/#:~:text=Recruitment%20refers%20to%20the%20process,a%20job%20in%20the%20organization (accessed January 25, 2022).

[B52] Tavana M. Mousavi H. Nasr A. K. Mina H. (2021). A fuzzy weighted influence non-linear gauge system with application to advanced technology assessment at NASA. Expert Syst. Appl. 182, 115274. 10.1016/j.eswa.2021.115274

[B53] Torre Olmo B. Cantero Saiz M. Sanfilippo Azofra S. (2021). Sustainable banking, market power, and efficiency: effects on banks' profitability and risk. Sustainability 13, 1298. 10.3390/su13031298

[B54] Valizadeh Gamchi F. Gürünlü Alma Ö. Arabi Belaghi R. (2019). Classical and Bayesian inference for Burr type-III distribution based on progressive type-II hybrid censored data. Math. Sci. 13, 79–95. 10.1007/s40096-019-0281-9

[B55] WallStreetMojo (2022). Importance of Ratio Analysis. Available online at: https://www.wallstreetmojo.com/importance-of-ratio-analysis (accessed January 25, 2022).

[B56] Wikipedia (2022a). The City Bank. Available online at: https://en.wikipedia.org/wiki/The_City_Bank (accessed January 22, 2022).

[B57] Wikipedia (2022b). Training and Development. Available online at: https://en.wikipedia.org/wiki/Training_and_development (accessed January 25, 2022).

[B58] Yasmin S. Alam M. K. Ali F. B. Banik R. Salma N. (2021). Psychological impact of COVID-19 among people from the banking sector in Bangladesh: a cross-sectional study. Int. J. Mental Health Add. 20, 1485–1499. 10.1007/s11469-020-00456-0 33495689 PMC7816746

[B59] Yusuf M. Ichsan R. N. (2021). Analysis of banking performance in the aftermath of the merger of bank Syariah Indonesia in Covid 19. Int. J. Sci. Technol. Manage. 2, 472–478. 10.46729/ijstm.v2i2.182

[B60] Zhang L. Guo X. Lei Z. Lim M. K. (2019). Social network analysis of sustainable human resource management from the employee training's perspective. Sustainability 11, 380. 10.3390/su11020380

[B61] Zhang Y. (2006). Content Analysis (Qualitative, Thematic). Available online at: http://www.ils.unc.edu/yanz/content20%analysis.pdf (accessed December, 2006).

[B62] Zhong Y. Li Y. Ding J. Liao Y. (2021). Risk management: exploring emerging Human Resource issues during the COVID-19 pandemic. J. Risk Financial Manage. 14, 228. 10.3390/jrfm14050228

[B63] Zupok S. (2017). Innovations as an element of building value for the client-RCGW SA case study in Tychy. J. Manage. Finance 15, 375–387. Available online at: http://www.wzr.ug.edu.pl/.zif/4_28.pdf

